# Predictive Processing in Sentence Reading: Semantic prediction but not phonological facilitation in deaf readers

**DOI:** 10.1016/j.cognition.2026.106703

**Published:** 2026-09-04

**Authors:** E. Gutierrez-Sigut, M. Perea, M. Vergara-Martinez, M. MacSweeney, K. Rowley, I. Saenz

**Affiliations:** University of Essex; University of Valencia; University College London

**Keywords:** Deaf readers, semantic prediction, phonological facilitation, reading, cochlear implant, bilingualism, deafness.

## Abstract

This study investigated whether deaf readers of English predict semantic and phonological information during sentence reading in the same way as hearing readers. Using a self-paced moving-window paradigm, we tested four groups who were matched on reading ability and English vocabulary knowledge: deaf readers without cochlear implants (CIs), deaf readers with CIs, hearing native readers of English (HN), and hearing non-native readers of English (HNN). Participants read sentences ending in one of five target types: congruent highly predictable words, congruent less predictable words, incongruent words, pseudohomophones of the highly expected word, or orthographic-control pseudowords. Semantic prediction was assessed by comparing reading times for congruent endings in high- and low-cloze contexts, and semantic congruency was assessed by comparing congruent and incongruent endings. Phonological contextual facilitation was assessed by comparing pseudohomophones with orthographic controls. All groups showed sensitivity to semantic incongruence, although Deaf-CI readers showed weaker evidence of semantic prediction, suggesting weaker reliance on predictive mechanisms during reading. In contrast, phonological facilitation was observed only in the HN group, despite the groups being matched on reading ability, suggesting that phonological facilitation during sentence reading may depend on hearing-native-like phonological representations. These findings support the view that skilled reading can be achieved through different routes. While semantic prediction appears to be broadly available across reader groups, phonological facilitation from an expected word form may depend on native-like, automatically engaged spoken-language phonological representations.

## 1 Introduction

Reading comprehension requires the reader to derive sentence meaning by first accessing the meanings of individual words and their syntactic relationships and then integrating this information into a coherent representation of the unfolding discourse (for overviews, see Mayer & Trezek, 2018; Perfetti & Stafura, 2014). In addition, readers actively generate predictions about upcoming words (for a recent review, see Wong et al., 2025), which can explain how real-time reading comprehension happens so quickly and effortlessly (Ferreira & Chantavarin, 2018; Kuperberg & Jaeger, 2016; Lupyan & Clark, 2015; Pickering & Gambi, 2018). Predictive processing, supported by contextual cues and the reader’s prior knowledge, facilitates word recognition and integration of meaning into the unfolding discourse. Huettig et al. (2022) conceptualise prediction as the preactivation of any piece of linguistic structure before its possible appearance in the input. This preactivated information can then interact with subsequent input and with other predictions, facilitating or interfering with processing. In the current study, prediction is operationalised as facilitation, reflected in faster reading times, of processing expected words during sentence reading for comprehension when compared to less predictable or incongruent words due to contextual predictability. Numerous studies have shown that predictable words are processed more efficiently than less predictable words: they elicit shorter fixation times and higher skipping rates (Ehrlich & Rayner, 1981; Kliegl et al., 2004; Luke & Christianson, 2016; Staub, 2015), smaller neural responses (e.g., DeLong et al., 2005; Kutas & Hillyard, 1984; Wlotko & Federmeier, 2015), and faster recognition times (e.g., Frisson et al., 2005; Staub, 2015).

While prediction during reading has been widely studied in hearing readers, it remains unclear whether and how these mechanisms operate in deaf readers. In the present study, we used a self-paced moving window reading task to investigate lexical prediction during reading comprehension in deaf readers. We tested four groups with different language backgrounds and access to speech phonology: deaf readers, deaf readers with a cochlear implant (CI), hearing native speakers of English, and hearing non-native speakers of English reading in their second language. These differing language backgrounds are expected to shape the nature of predictions in distinct ways, offering insight into both the mechanisms that support sentence reading in deaf people and the broader dynamics of predictive processing across diverse reader profiles.

### 1.1 Deaf readers

Although some deaf people become skilled readers, many find reading to be challenging. Older studies estimated that deaf students left school with reading levels roughly equivalent to those of hearing 9- to 10-year-olds (Conrad, 1977; Sánchez & García-Rodicio, 2006; Traxler, 2000; Wauters et al., 2006). More recent work suggests that, while outcomes are improving for some groups (Couvee et al., 2024; 2025), the reading levels of many deaf children remain at the lower end of the hearing norms (e.g. lower 20% reading comprehension in Couvee et al., 2025) and show substantial variability (Kyle & Cain, 2015; Mathews & O’Donnel, 2020). Thus, a gap remains particularly as children progress through school and encounter more complex texts, which can hinder success in further or higher education.

The low reading proficiency observed in some deaf people is often explained in terms of underspecified print-to-sound mappings and the accompanying reduced reliance on spoken phonology during word decoding (Perfetti & Sandak, 2000). It is widely believed that poor phonological decoding acts as a bottleneck to further reading comprehension. However, recent evidence from deaf adult readers shows that the picture is more complex. First, a clear link between phonological processing and reading skill has not been consistently found in deaf signing adults (for reviews, see Emmorey, 2020; Emmorey & Lee, 2021; Gutierrez-Sigut et al., 2017; 2018). Second, access to meaning through visual and orthographic cues may predict reading proficiency, in deaf skilled readers, to a much greater extent than phonological processing (Emmorey & Lee, 2021; Gutierrez-Sigut et al., 2019; Meade et al., 2020). For example, Gutierrez-Sigut et al. (2019) found that stronger interactions between orthographic and lexico-semantic processing during visual word recognition were associated with better reading skill in deaf readers.

While many prior studies have focused on isolated-word recognition (for reviews, see Emmorey, 2020; Emmorey & Lee, 2021), increasing attention has been paid in recent years to sentence-level comprehension mechanisms. This shift is important because tasks that involve full sentences and require participants to read for meaning more closely approximate natural reading processes than tasks that use single-word stimuli (e.g., the lexical decision task). This distinction is critical because the cognitive mechanisms engaged during word recognition are highly sensitive to task demands and can vary depending on whether the task emphasizes form-based or meaning-based processing (Adams & Kiefer, 2012; Kiefer et al., 2012; Meade et al., 2019). For instance, Rowley (2018) adapted the visual-world paradigm for deaf participants by replacing auditory stimuli with written words. Words were presented in the centre of the screen with the target and distractor picture surrounding it.

They found that both deaf and hearing readers accessed phonological information in pseudohomophones (e.g., KOTE for COAT) during lexical decision over written items presented in isolation, but only hearing readers showed a phonological effect in a word-picture matching task that required semantic processing (see Rowley et al., 2025). Such task-dependent variation in phonological effects may help explain why previous studies, which have used a range of tasks, have not consistently linked phonological processing to reading ability in deaf adults (Emmorey, 2020; Emmorey & Lee, 2021; Gutierrez-Sigut et al., 2017, 2018).

Sentence comprehension draws on multiple sources of information, including orthographic, semantic, contextual and speech-phonology information. The extent to which deaf and hearing readers draw on these sources of information is likely to differ. In addition, deaf signers may also draw on sign-language or fingerspelling representations during reading. Within the deaf population, the relative contributions of the different sources of information are likely to vary with type of written script, methodology, task demands, as well as participants’ language experience and reading skill. Previous research on sentence reading in deaf adults suggests systematic differences in the mechanisms used by deaf and hearing readers during comprehension. In particular, deaf readers appear to rely more on visual-orthographic information, direct access from orthographic forms to word meanings (semantics), and sentence-level contextual information than hearing readers, whereas the contribution of phonological information from words appears to be more variable. A small number of eye-tracking studies on skilled readers have suggested greater processing efficiency on specific eye-movement measures during sentence reading in deaf compared to hearing skilled readers, including greater word skipping and shorter fixation durations (Bélanger & Rayner, 2015), likely reflecting enhanced visual linguistic processing and a greater reliance on word length and predictability rather than phonological cues (Bélanger et al., 2013; Bélanger & Rayner, 2013, 2015; Gutierrez-Sigut et al., 2019; 2022). Thierfelder et al. (2020) found that deaf readers of written Chinese relied primarily on visual orthographic information to access word meanings during sentence reading, with phonological (homophonic) errors having a late effect overall—although note that the phonological effect emerged earlier during processing in highly predictable than low predictability sentences. Other sentence-reading studies show early access to parafoveal semantic information in deaf readers of Chinese and indicate that the timing and nature of phonological activation vary with language experience and reading ability (Yan et al., 2015; Lan et al., 2026; Thierfelder, 2024). For signers, written words may also activate sign-language representations, which could provide another form of phonological (i.e. sign phonology) mediated form-processing (Pan et al., 2015; Saunders et al., 2025). Neuroimaging studies show that although both deaf and hearing readers activate core left-hemispheric language regions during sentence comprehension, deaf readers also recruit visual and right-hemisphere areas and exhibit distinct connectivity patterns (Emmorey, 2020; Emmorey et al., 2025; Emmorey & Lee, 2021; Emmorey et al., 2013; Glezer et al., 2025); this right-hemisphere involvement appears adaptive in skilled deaf readers and is consistent with a reading profile that relies less on phonology and more on orthographic-semantic processing, which is more bilaterally distributed than phonological decoding. Behavioural work converges with this view, for example, Fischler (1985) investigated word integration in deaf and hearing college-level readers using a lexical decision task with sentence fragments followed by congruent or incongruent targets. Deaf readers relied more strongly on contextual cues than hearing readers. Deaf readers also appear to rely more heavily than hearing readers on key words and semantic cues when resolving syntactic-semantic conflicts (Domínguez & Alegria, 2010).

At the same time, this pattern is not uniform across all deaf readers. Bélanger and Rayner (2013) showed that the use of contextual information varies with reading skill, suggesting that increased reliance on context may reflect skill level rather than deafness per se. Similarly, Mehravari et al. (2017) found that the most skilled deaf readers were more sensitive to semantic errors in sentences, whereas the most skilled hearing readers were sensitive to grammatical errors. Hirshorn et al. (2015) further showed that the best predictor of reading comprehension differed across deaf readers: free recall was the strongest predictor for deaf native signers, whereas phonological knowledge best predicted reading comprehension in oral deaf individuals. Taken together, these findings suggest that semantic processing plays a central role in sentence reading comprehension for deaf adults, whereas the contribution of phonological processing remains less clear and may vary with language experience (Hirshorn et al., 2015; Mayberry et al., 2011). In the present study, we ask whether at comparable levels of reading skill deaf readers use contextual cues to facilitate semantic and phonological processing during sentence reading and whether these effects relate to reading skill and language experience.

One language-experience factor that could affect the use of phonological information during reading is having a cochlear implant (CI). CIs increase access to the auditory component of speech and, for many deaf children, support spoken language development (e.g., De Raeve, 2010; Marschark et al., 2007; Vermeulen et al., 2007). Post-implantation, users often show marked gains in sound and speech detection, speech perception, and speech intelligibility (AlSari, 2024; Malhotra et al., 2021). More than half of children implanted by age 5 have been reported to achieve spoken language skills similar to hearing age-peers in verbal reasoning, narrative ability, and vocabulary (AlSari, 2024; Svirsky et al., 2000). Because spoken language development is a fundamental factor in explaining reading skills in hearing children, it has often been assumed that the increased access to speech provided by a CI may also support literacy in deaf readers (see, e.g., Mayer and Trezek, 2024). Extending this logic, if CI users develop stronger spoken-language representations, one possibility is that they may be more likely to recruit phonological information during reading, including in predictive processing. However, this remains a hypothesis to be tested. Indeed, findings on reading outcomes in children with CIs are mixed. While some studies report age-appropriate reading levels among children with CIs (e.g., Archbold et al., 2008; Mayer & Trezek, 2018; Mayer et al., 2016), other research suggests that their performance in areas such as word decoding, reading comprehension, and related skills such as vocabulary or phonological awareness is similar to that of deaf children using hearing aids (e.g., Connor & Zwolan, 2004; Fitzpatrick et al., 2012). If phonological awareness and word decoding skills do not consistently resemble those of hearing readers, then the assumption that CI users read using the same mechanisms as hearing readers is not straightforward.

Whether CIs facilitate the use of phonological information from words during reading remains unclear, particularly because research with deaf adult readers who received a CI during development is very limited. Next, we review evidence regarding semantic and phonological prediction in hearing readers, which informs the predictions for the present study.

### 1.2 Semantic prediction

Semantic prediction refers to the process by which the preactivation of semantic features, based on the prior context of a sentence, facilitates the processing of a word’s meaning when it is encountered. Electrophysiological studies using rapid serial visual presentation (RSVP) have consistently shown reduced N400 amplitudes for predictable words, compared to unpredictable words, reflecting facilitated semantic access (DeLong et al., 2005; Federmeier & Kutas, 1999; Kutas & Hillyard, 1984; Wlotko & Federmeier, 2015; although see Ito et al., 2017a). As these effects are robust across different levels of sentence constraint and presentation rates, semantic prediction is assumed to proceed automatically during reading comprehension (Ito et al., 2016; Thornhill & Van Petten, 2012).

Self-paced reading, which allows a more naturalistic timing than RSVP, also supports the use of semantic prediction in hearing readers. Hearing readers tend to show slower reading times for unexpected than expected words in highly constraining contexts (Payne & Federmeier, 2017) such as “*Every morning, she spread butter on her* …”, which strongly predicts the ending “*toast*”. High sentence constraint is typically indexed by high-cloze probability (i.e. the likelihood that a specific word will be supplied by participants when asked to complete a sentence). Self-paced reading times are influenced by both the predictability of the word within the sentence and semantic relatedness of possible sentence completions (Hintz et al., 2016; Roland et al., 2012). Payne and Federmeier (2017) combined self-paced reading with electrophysiological recordings and found that unexpected words in strongly constraining contexts led to slower reading times and a larger N400. Taken together, these findings suggest that semantic facilitation may result from the preactivation of semantically compatible words, either through spreading activation within the lexicon or through simultaneous activation of multiple candidates consistent with contextual constraints (e.g., Roland et al., 2012).

While there is general agreement that hearing readers engage semantic prediction to a degree during reading, previous evidence suggests that its use is flexible and shaped by text characteristics, individual differences, and linguistic background. Regarding text characteristics, Carter and Hoffman (2024) found that predictability effects are stronger in coherent discourse, but diminish when coherence drops, indicating that readers adjust predictive processing based on contextual reliability. Regarding individual variability, while some studies report stronger predictability effects in skilled readers (Ng et al., 2017; Steen-Baker et al., 2017), others find larger effects in less proficient readers, particularly in those with slower reading speeds (Ashby et al., 2005; Slattery & Yates, 2018). Vocabulary knowledge has also been linked to faster integration of contextual information (Guerra & Kronmüller, 2020), as well as some cognitive profiles. Milin et al. (2017) demonstrated that semantic prediction is shaped by cognitive profile: readers varied in their reliance on top-down semantic generalizations, suggesting that some readers are more likely to exploit semantic context for prediction and integration.

Finally, linguistic background also shapes semantic prediction. Native speakers show clear semantic prediction effects, whereas readers of a second language (L2) typically exhibit weaker or absent predictability effects, likely because lower L2 proficiency limits activation of context-relevant information (Ito & Pickering, 2021). For example, Ito et al. (2017b) found that semantically related words lead to an N400 reduction in L2 speakers, but the reduction was not directly dependent on the cloze probability of the predictable word. This finding suggests that the facilitation may reflect the ease of integration of semantically related words into the context, rather than a genuine prediction based on probability, which is typically seen in L1 processing (Ito et al., 2016; Ito et al., 2017b; Van Petten & Luka, 2012).

### 1.3 Phonological prediction and facilitation

While semantic prediction has been widely studied, fewer studies have examined whether readers predict the specific form of words, orthographic or phonological, during sentence reading. While many studies have focused on orthographic preactivation (see, e.g.,, DeLong et al., 2005) or have used the broader term “form prediction” to encompass both orthography and phonology (see, e.g.,, Ito et al., 2016), relatively few have isolated phonological prediction as a distinct phenomenon.

In an eye-tracking study, Rayner, Pollatsek, and Binder (1998) found that highly constraining contexts can reduce disruption from phonologically appropriate spelling errors (pseudohomophones), concluding that readers may relax orthographic verification when the sound of the input matches strong top-down expectations. However, Daneman and Reingold (2000) argued that phonological activation during reading can be weaker and/or delayed relative to direct orthographic access to meaning, with phonological effects appearing only in highly predictable contexts. In an early electrophysiological study of phonological prediction, Connolly et al. (1995) investigated whether sentence endings that were phonologically related to a predictable word would elicit not only an N400 response, but also earlier electrophysiological components previously observed in auditory studies. The experiment contrasted sentence-final words across four conditions: (1) fully congruent (e.g., Ray fell down and skinned his *knee*); (2) phoneme match–semantic mismatch, where the word began with the initial phoneme of the expected ending but was semantically anomalous (e.g., They sat together without saying a single *worm* [expected: word]); (3) phoneme mismatch–semantic match, where the word was semantically related but began with an unexpected phoneme (e.g., The pigs wallowed in the *pen* [expected: mud]); and (4) phoneme mismatch–semantic mismatch, involving words that were both phonologically and semantically inappropriate (e.g., The winter was very harsh this *allowance* [expected: year]). They concluded that phonological prediction does not occur during sentence reading because the phoneme mismatch–semantic match and phoneme match–semantic mismatch (*mud* for *pen*) conditions did not differ in the early window, and the N400 was insensitive to phonological mismatch. However, Newman and Connolly (2004) used a similar design but presented high-cloze sentences ending in a congruent word, an incongruent word, a pseudohomophone (e.g., *The ship disappeared into the thick phog* [fog]), or an incongruent pseudoword (e.g., *The gas station is about two miles down the bole* [road]). They found a reduced N400 in the pseudohomophone condition, suggesting that phonological congruency facilitated semantic integration even when orthographic expectations were violated.

More recent findings across methodologies also suggest that skilled hearing readers routinely preactivate form (to a large extent orthographic) information during sentence reading, facilitating fluent comprehension (see, e.g.,, Amenta et al., 2023; DeLong et al., 2019, 2021; DeLong et al., 2005; Ito et al., 2016; Tiffin-Richards, 2024; Yan et al., 2020; Yan et al., 2024). Phonological prediction, however, appears to be restricted to sufficiently constraining contexts, weaker than semantic activation, and highly dependent on experimental setup. Ito (2024) conducted a meta-analysis of 20 visual-world eye-tracking studies to evaluate the robustness and timing of phonological prediction. The results revealed a small but reliable effect: participants fixated more on phonological competitors than other distractors before the target word was mentioned, indicating that phonological forms can be preactivated under highly predictive conditions. This phonological prediction effect is not only sensitive to contextual constraint, but also to experimental features. For example, form-related N400 and late positive component (LPC) reductions were observed at a 700 ms stimulus onset asynchrony (SOA), but not at 500 ms (Ito et al., 2016) showing that sufficient processing time is crucial (but for a different view see also DeLong et al., 2019, 2021). These findings suggest that phonological preactivation may be more limited and slower to emerge than semantic prediction, supporting a hierarchical spread from meaning to form. Huettig et al. (2022) proposed that phonological prediction is generally weaker than semantic prediction because phonological cues (i.e. how earlier words sound) do not accumulate as the sentence unfolds as semantic cues do.

Language background also influences phonological prediction, with evidence from L2 readers remaining mixed. Non-native (L2) readers often fail to show form effects under conditions where native speakers do (Ito et al., 2018) and tend to rate the same items as less predictable (Ito et al., 2017b), suggesting that stronger contextual constraints may be needed to elicit similar effects in L2 readers. Slower and more effortful processing in L2 is likely to further hinder timely prediction (see, e.g,, Wlotko & Federmeier, 2015).

### 1.4 The current study

The present experiment aims to shed light on whether comparable levels of reading skill and sentence comprehension can be achieved through the same or through different online processing mechanisms. In particular, we ask whether readers who differ in hearing status and access to speech phonology use contextual information to predict semantic and phonological information during sentence reading, and whether the use of these mechanisms relates to reading skill and language experience.

The primary focus was on readers who were severely or profoundly deaf from early in life and did not have a cochlear implant. Within this group, written English can be considered a second language for participants who sign. To examine how linguistic proficiency and language background influence prediction in deaf readers, we also included two hearing comparison groups: hearing native speakers of English and hearing non-native English speakers that were reading in their second language. In addition, as previously outlined, access to speech can vary widely depending on whether severely to profoundly deaf people have a CI. To test whether increased access to speech phonology via CI facilitates the use of phonological information during sentence reading, we also included a group of deaf readers with CI. All groups were matched for reading skill and vocabulary knowledge with the group of deaf readers without CI. For each group separately, we also examined whether the semantic and phonological effects were related to reading ability, vocabulary-based English proficiency, and orthographic and phonological skills.

In this study, we maximised the likelihood of detecting phonological facilitation effects compatible with phonological prediction by using highly constraining sentence contexts (cloze probability > 80%) and pseudohomophones of the expected word rather than real words that elicit their own lexical access process as in Ito et al. (2016). The pseudohomophones that we used—and their matched orthographic controls—have previously been shown to elicit phonological effects in single-word priming studies (specifically in Blythe et al., 2018; Lukatela et al., 1998; Lukatela & Turvey, 2000; Rastle & Brysbaert, 2006). We also used self-paced reading to address limitations of fixed presentation rates in RSVP and to assess whether phonological facilitation emerges under more naturalistic timing conditions.

Our hypotheses were:

1. Semantic prediction: In line with previous research with hearing (for review see Wong et al., 2025) and deaf readers (Bélanger & Rayner, 2013; for review see Emmorey & Lee, 2021), we expected both semantic congruency and semantic prediction effects in all groups, reflected in shorter reading times for congruent than incongruent and for congruent than low-cloze sentence endings respectively. As in Ito et al. (2017b) and Ito and Pickering (2021), non-native speakers of English may show weaker semantic prediction effects. We therefore expected larger effects for hearing native speakers compared to non-native speakers.
2. Phonological facilitation: Based on previous evidence of form preactivation during reading in hearing readers (Dimigen et al., 2011; Ito, 2024), reduced reliance on phonological processing in deaf readers (Bélanger et al., 2013; Emmorey, 2020; Emmorey & Lee, 2021; Gutierrez-Sigut et al., 2022; Gutierrez-Sigut et al., 2017; Gutierrez-Sigut et al., 2019) and weaker or non-existent form prediction in L2 readers (Ito et al., 2017b; Ito et al., 2018; Wlotko & Federmeier, 2015) we expected phonological facilitation during sentence reading in hearing native speakers but not in the deaf readers without CI or the hearing non-native speakers. Whether deaf participants with a CI will show an effect of phonological facilitation was an open question.

## 2 Methods

### 2.1 Design

Participants performed a moving window self-paced sentence reading task (as in Cates et al., 2022) online using their own computers. The sentences could end in a high-cloze probability word, a low-cloze congruent word, a semantically incongruent word, or one of two types of pseudowords—either a pseudohomophone of the high-cloze probability word or an orthographic control that shared visual properties with the pseudohomophone (see materials for examples). Besides this experimental task, participants completed the following language tasks:

#### 2.1.1 Vernon Warden reading test (Hedderly, 1996).

A standardised cloze-style sentence completion test in which participants have a maximum of 10 minutes to complete as many items as possible from a total of 42 items. The items consist of incomplete sentences and 5 possible response options from which the participant must choose the one that correctly completes the sentence. The items increase in difficulty as the test progresses. The score was calculated as the number of correct responses, with a penalty of one point for every five incorrect responses. This test does not assess discourse-level reading comprehension.

#### 2.1.2 LEXTALE (Lemhöfer & Broersma, 2012).

A standardised assessment of English word knowledge. Participants judge the lexical status of 60 letter strings (40 English words and 20 non-words). After a 250 ms fixation cross and a 100 ms blank, the letter strings are displayed one at the time, in a fixed order, on the centre of the screen until the participant responds. Task duration is approximately 2-3 minutes. Scores were calculated as averaged percentage correct, following Lemhöfer and Broersma (2012): the percentage correct for word items and the percentage correct for nonword items were computed separately and then averaged.

#### 2.1.3 Phonological decoding skill (Olson et al., 1994).

Three non-words are presented side by side (e.g. *nite / kile / hote*). Participants must select as quickly as possible which non-word sounds like a real word (e.g., *nite – night*). There are 5 practise trials followed by 60 trials. The three options remain on screen until the participant’s response or 10 seconds have elapsed. The task duration is approximately 5 minutes. Participants receive feedback (correct answers) only during practise trials and not during the experimental trials. The score was calculated as the percentage of correct responses.

#### 2.1.4 Orthographic decoding skill (Olson et al., 1994).

Two items are presented side by side (e.g. *room/rume*), only one of them is a correctly spelled word. Participants must select as quickly as possible which is the correct spelling. There are 5 practice trials followed by 80 trials. The two options remain on screen until the participant’s response or 10 seconds have elapsed. The task duration is approximately 5 minutes. Participants receive feedback (correct answers) only during practice trials and not during the experimental trials. The score was calculated as the percentage of correct responses.

### 2.2 Participants

#### 2.2.1 Deaf non-CI

Twenty-five deaf readers, without a CI, were recruited for this study. Data from one of the deaf participants was not recorded due to a technical issue. The average age of the remaining 24 participants^1^ was 33 years (SD = 9.5; range 22 to 61) (10 female, 14 male), 21 reported being deaf between birth and age 3, and three participants reported having been diagnosed as profoundly deaf between ages 4 and 6^2^. One participant did not know British Sign Language (BSL), 12 were native signers, 6 were early signers (BSL AoA between 4 and 8 years old), and 5 were late signers (BSL AoA 9 years old and older)^3^. The participants were recruited through invitation due to having participated in previous research and via researchers’ personal contacts.

#### 2.2.2 Deaf-CI.

Fifty-one deaf participants with a cochlear implant (CI) also participated in this study. We selected 24 participants to match the deaf non-CI group on reading ability (LMM analyses for the larger groups are available in the supplementary materials). Participants were matched first on Vernon–Warden score, by selecting for each deaf reader the participant with the closest score from the large pool. Where more than one candidate was available, matching was refined using LEXTALE score. If a tie remained, age and then gender were used as further matching criteria. The average age of the subgroup of 24 participants was 27yrs (SD = 5.6; range 20 to 42). The selected 24 participants (12 female, 11 male, 1 non-binary) reported being severely or profoundly deaf before age 3 years, except for one who reported having been diagnosed between ages 4 and 6 years, and having their CI fitted at age 5 years. Their age of implantation varied from 0 to 31 years of age, with 18 participants reporting having had an implant before age 9 years, three participants reported having their implant fitted at ages 22, 28, and 31 years, and three participants did not answer this question. Four of the early-implanted participants reported that they no longer use their implants. One participant did not know British Sign Language (BSL), 12 were native signers, 8 were early signers (BSL AoA between 4 and 8 years old), 1 was a late signer (BSL AoA 9 years old and older), and 3 did not report when they started to sign. Participants were recruited through invitation due to having participated in previous research and via researchers’ personal contacts.

#### 2.2.3 Hearing Native Speakers of English (HN).

One hundred and eleven hearing participants who were native readers of English also participated in this study. A total of 24 participants (17 female, 7 male) were selected from this larger pool to match deaf readers reading ability (using the closest neighbour procedure described for Deaf-CI readers). Their average age was 31yrs (SD = 9.7; range 19 to 50). Participants were recruited through Prolific and via the participant’s database at the University of Essex.

#### 2.2.4 Hearing Non-native Speakers of English (HNN).

Eighty-three hearing participants that were non-native readers of English were also recruited. All HNN participants were proficient in English, and they had passed the necessary English exams to be admitted to university. A total of 24 participants (19 female, 5 male) were selected from this larger pool to match deaf readers reading ability (using the closest neighbour procedure described for Deaf-CI readers). Their average age was 31yrs (SD = 13; range 20 to 60). Five of the participants reported Mandarin Chinese as their first language; 4 reported Cantonese; and the rest had learned Portuguese, Romanian, Malayalam, Albanian, Hindi, Polish, Konkani, Norwegian, and French as their first language. Among the 24 participants, four learnt English between 0 and 2 yrs, eleven between 3 and 5yrs, five between 6 and 8 yrs, three between 9 and 11, and finally one was over 15 yrs when they learnt English. All reported to have learnt English as an L2 in restricted environments/hours of the day while immersed in their L1 environment. All participants were recruited through Prolific and via the participant database at the University of Essex.

This research was approved by the University of Essex Science and Health ethics subcommittee 3 (ETH2021-0196). All participants received either course credits (if students) or 10.00 GBP per hour per their participation and gave informed consent.

**Figure 1:**
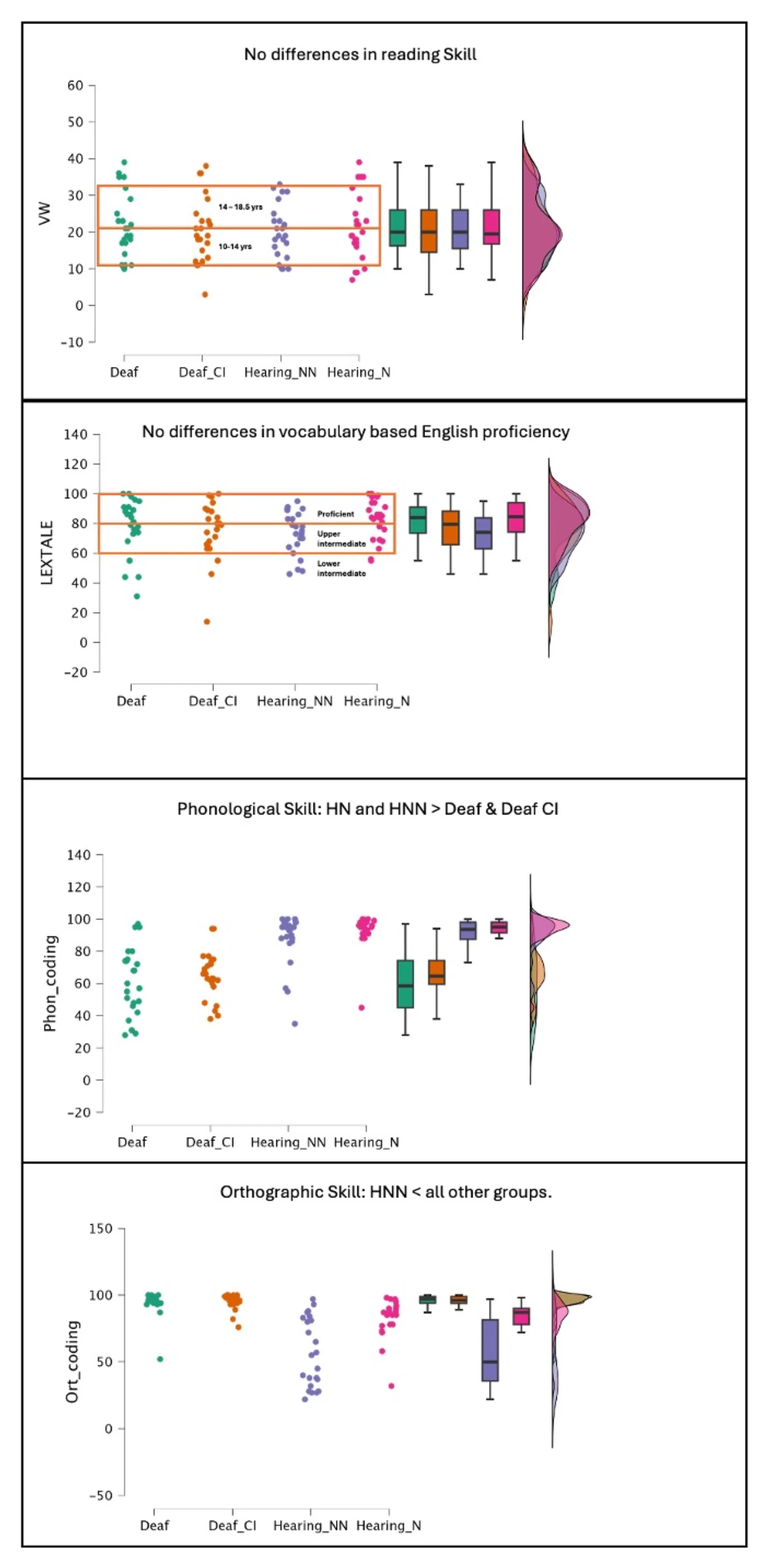
Distribution of scores of each group (Deaf, Deaf CI, HNN, and HN from left to right in each graph) in reading skill measured with the Vernon Warden reading test (top left), English vocabulary measured with LEXTALE (top right), phonological skill (bottom left) and orthographic skill (bottom right). For reference, the orange lines in the reading skill plot show raw scores corresponding to 10 to 14 yr and 14 to 18.5 yr reading ages. For reference, the orange lines in the vocabulary-based English proficiency test show the scores corresponding to proficient, upper intermediate and lower intermediate.

ANOVAs were run to compare the groups on reading skill, English vocabulary (LEXTALE), and phonological and orthographic skills [Anova [*skill of interest*] ~ Group, pstHocCorrectionHolm = TRUE, postHocTerms = ~ Group]. These analyses showed that the four groups were matched in reading ability (all *p*s >.1), and English vocabulary (all *p*s >.2). Figure 1 shows the distribution of scores for each group in each of the skills. The hearing HN and HNN groups did not differ in phonological coding skill (*p* >.1), the deaf and deaf CI groups did not differ in phonological skill (*p* >.1), but both deaf groups had a significantly lower score in the phonological skills task than the HN and the HNN groups (all *p*s <.001). The HNN group had a significantly lower score than all other groups in the orthographic skill task (all *p*s <.001), the difference between the deaf group and the HN group approached significance (*p* = .069) with the deaf readers having higher scores than the HN readers (94.7% and 82.7% correct respectively). The difference between the deaf CI group and the HN group also approached significance (*p* =.050) with the deaf CI readers having higher scores than the HN readers (95.2 and 82.7 % correct respectively.)

**Figure 2:**
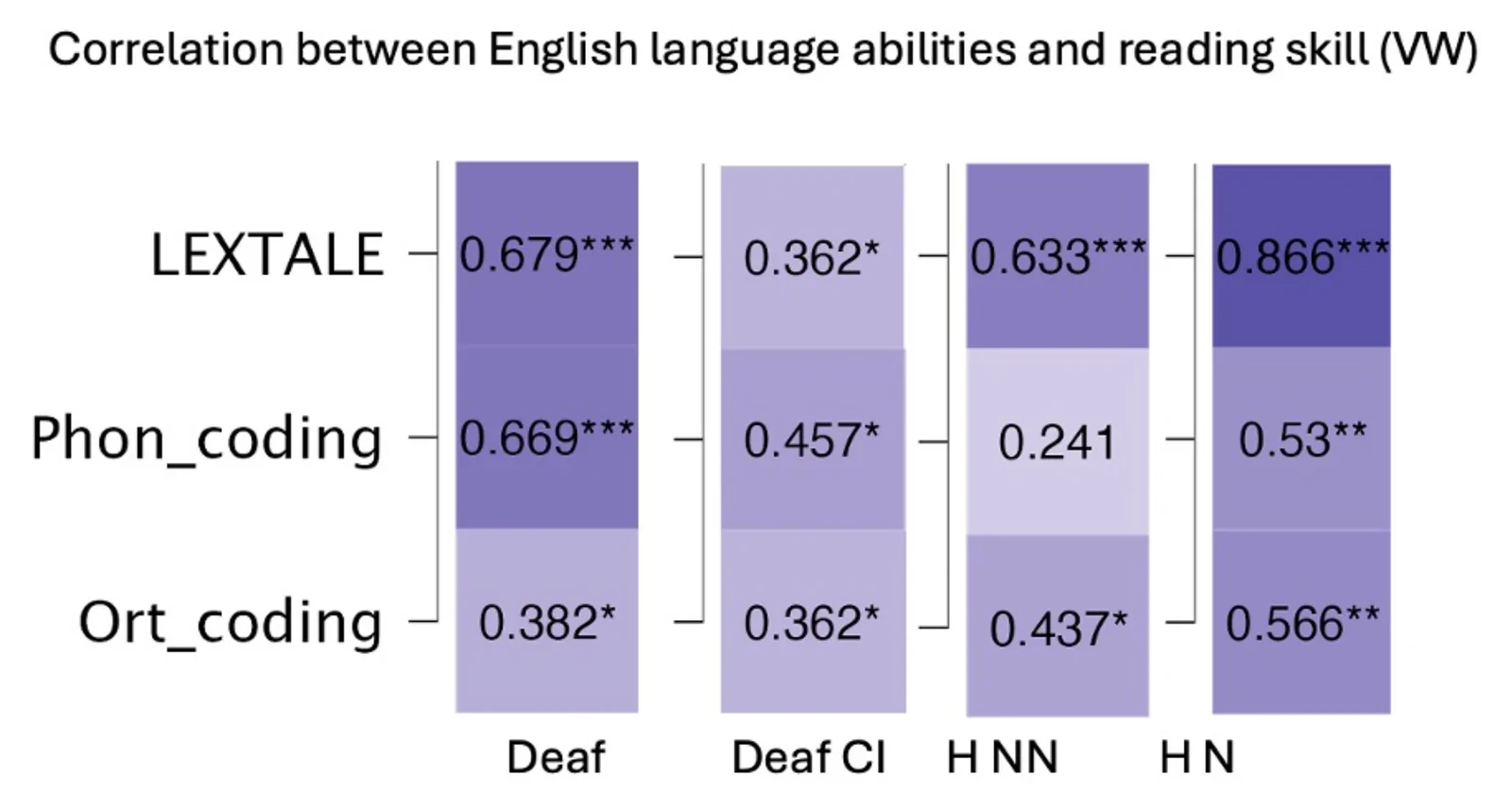
Correlations between reading skill and vocabulary-based English proficiency measured with LEXTALE (top row), phonological skills (middle row), and orthographic skill (bottom row) for each group (Deaf, Deaf CI, HNN, and HN from left to right). The figure shows the r values, significance levels are indicated with * (* = *p* < .05, ** = *p* < .01, and *** = *p* < .001).

Figure 2 shows the correlations between reading ability and the other three skills, measured separately for each group. For all groups, reading ability was correlated with English vocabulary and with orthographic skill (all *p*s < .033). For the Deaf, Deaf CI and HN groups, reading ability was also significantly correlated with phonological skill (all *p*s < .012). This correlation was not significant for the HNN group (all *p*s = .121).

### 2.3 Materials

We created 136 high-cloze probability sentences in which the ending was manipulated. The sentence ending was either 1) a congruent, highly expected word; 2) a real word that was incongruent with the sentence; 3) a pseudohomophone (a pseudoword that sounded like the congruent and highly expected ending); or 4) an orthographic control (a pseudoword whose orthography matched the pseudohomophone). For 7 of the sentences the critical word was not the last word in the sentence (e.g. “*Hugo wants to buy a car but needs to save some* ***money*** *first*”) because during materials development and validation native speakers judged these sentences as incomplete or insufficiently natural without an additional ending. Cloze probability was assessed on the sentence frame up to the target word, so this did not affect the predictability manipulation itself. Participants also read 34 low cloze probability sentences with congruent endings. The semantic effect was operationalised as the comparison between congruent high-cloze and congruent low-cloze, as well as between congruent high-cloze and incongruent words. The phonological effect was operationalised as the contrast between pseudohomophone and orthographic control. For example (target item in bold):

1) **Congruent**: Monica left the teabag in the teapot and allowed the tea to **brew**.
2) **Incongruent**: Monica left the teabag in the teapot and allowed the tea to **won**.
3) **Low Cloze:** The lamp was broken at the **base**.
4) **Pseudohomophone**: Monica left the teabag in the teapot and allowed the tea to **brue**.
5) **Orthographic Control**: Monica left the teabag in the teapot and allowed the tea to **bree**.

There were 20 filler sentences (e.g., “Victoria was worried that her dyed hair colour would **fade**”), 10 of them ending in a semantically incongruent word (e.g., “April ran her errands during her lunch **fire**”). Therefore only 36% of the sentences ended in a pseudoword and 23% of the sentences ended in a semantically incongruent word. These fillers were added to discourage strategies based on the proportion of pseudowords and incongruent items.

The process of creating the sentences was as follows:

We selected stimuli previously used in pseudo-homophone priming experiments that found a phonological priming effect in hearing readers from a pool of published studies (Blythe et al., 2018; Lukatela et al., 1998; Lukatela & Turvey, 2000; Rastle & Brysbaert, 2006). The pseudohomophone sounded like its corresponding base word (e.g., brew - brue). The length and degree of visual similarity between the pseudohomophone and the base word were not controlled, so some items had high visual similarity (e.g., car - kar) while others had low visual similarity (e.g., roars - rauze). The orthographic control pseudowords were matched to the pseudohomophones in length, bigram frequency, and Levenshtein distance to the base congruent word (e.g., pseudohomophone: brue and orthographic control: bree for the base word brew).

Sentences were created with the aim for the base word to be highly predictable and then validated. First, a group of 100 psychology students rated the correct sentences for grammaticality (Is this sentence grammatically correct?) and how natural they were (Is this sentence natural? Is it something that you would say or hear?). Only sentences that scored 7 or more on a 1-10 grammaticality and naturality scale were selected. Second, a different group of 100 psychology students were given the sentence without the key word and were asked to type which word they thought would complete the sentence best. The 136 experimental items that had 80% or more agreement on the key word were selected as high-cloze experimental items. Items that had lower agreement (60% and lower) were selected as low-cloze experimental items (n = 36) or fillers (not analysed n = 20). All experimental sentences had a Flesch-Kincaid score of 4.1 which is a basic reading level corresponding to elementary school (9 to 10 yrs).

### 2.4 Procedure

Participants completed the tasks at home on their own computers via the Psytoolkit (Stoet, 2017) platform. Upon receiving the information sheet and the link to the study all participants were offered a Zoom meeting or communication via smartphone text, with a native signer for deaf signers, to clarify instructions and answer questions at any point during the study. Participants were asked to start the tasks only when they had 1 hour free of commitments, and to switch off notifications and all other software running on their computers for the duration of the task. Psytoolkit was programmed to maximise its browser tab and adapt font size to the participant’s screen size. A small percentage of the hearing participants completed the tasks at a lab at the University of Essex. We did not observe differences in reading speed or percentage of correct responses between the participants in the lab and those at home.

Participants completed the self-paced reading, phonological skill, orthographic skill and LEXTALE tasks in a random order. The Vernon Warden reading test was the last task for all. In the experimental self-paced reading task, participants saw first a series of XXX on the screen that matched the length of the words in the sentence. They pressed the SPACE BAR to uncover each of the words. When one word was uncovered the previous was covered again. Participants were instructed to read normally to understand the content of the sentence. They were asked to press the space bar at their own reading speed, doing their best to understand what they were reading because they could not go back. After the last word of the sentence, they pressed the space bar to see the comprehension question. There were two response options (only one correct), and they used the mouse to click on the correct answer. The comprehension questions followed every sentence, referred to the first part of the sentence, and were only present to ensure task compliance. Participants could take breaks at any point after answering the question.

There were four counterbalancing groups (Latin square counterbalancing), so across participants, each sentence context was seen in all four conditions. Each participant only saw each of the 136 sentences in one of the four experimental conditions (congruent, incongruent, pseudohomophone, and orthographic control). All participants saw the same 34 low-cloze sentences and 20 fillers (not analysed). There were four practice sentences.

## 3 Results

**Figure 3:**
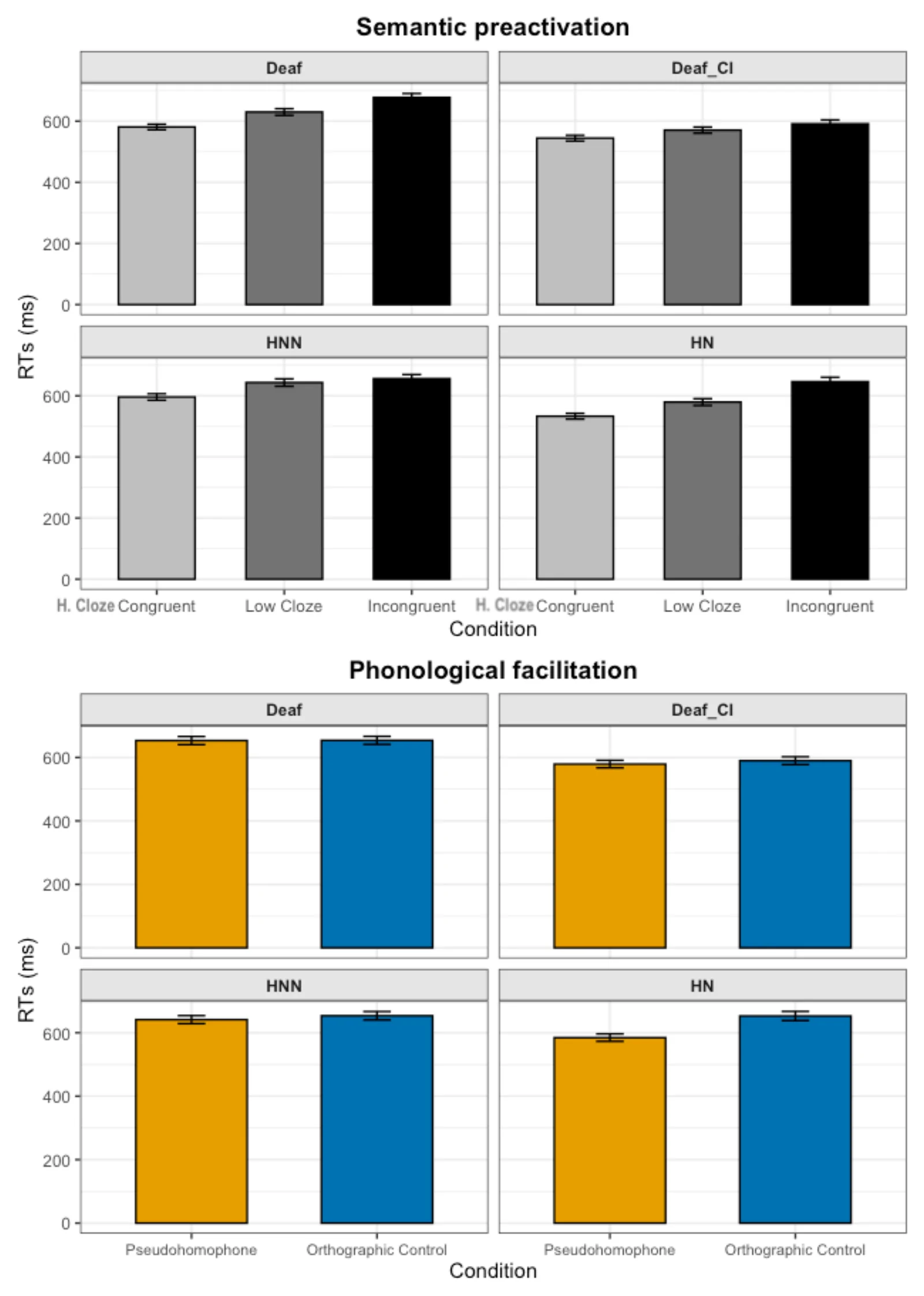
Average reading times in milliseconds for the experimental conditions: High Cloze Congruent (light grey), Low Cloze (dark grey), Incongruent (black), Orthographic control (blue) and pseudohomophone (orange) for each group separately (Deaf, Deaf CI, HNN, and HN). Error bars show SE.

We analysed reading times (RTs) to the target word. RTs below 250 ms and above 2500 ms (2.7%) were excluded from the analyses. Since the incongruent, pseudohomophone, and orthographic control conditions were designed to be not fully comprehensible, no RTs were excluded due to errors in the comprehension questions (see Keating & Jegerski, 2015). All participants engaged with the task and were highly accurate on the task. Mean accuracy was 93.6% (*SD* = 5.4) correct (Deaf = 92.9%, Deaf CI = 93.8%, HN = 95.3%, and HNN = 91.3%). There were no differences in accuracy to the comprehension questions between the groups, *F*(3, 90) < 1. Table 1 details the average reading times for each condition and group (see also Figure 3).

To analyse the RTs to the targets we fitted a generalized linear mixed-effects model (GLMM), in R (R Core Team, 2021) (version 4.4.1) using the glmmTMB package (Brooks et al., 2017). The model used a Gamma distribution with a log link to account for the positive skew of RT distributions (Lo & Andrews, 2015). We used the most complex model random-effects structure that converged (RT ~ Group * Type of ending + Age + Orthographic decoding + Phonological decoding + Length + Lexical Frequency + ONdensity + ON frequency + (1 + Type of ending || ID) + (1 | itemID)) following recommendations for maximal random-effects structures (Barr et al., 2013). The random effect structure comprised random intercepts and slopes for participants. Age was added as a participant-level covariate to account for potential differences in age distribution between the groups, Orthographic and Phonological Decoding skills were also added as participant-level covariates. Length, orthographic neighbourhood density, and orthographic neighbourhood frequency were added to model systematic lexical variability, while random intercepts for items captured residual item-to-item variability.

To test our hypothesis, we specified planned contrasts using the emmeans package (Lenth, 2023), estimated marginal means were computed on the response (reading-time) scale, Holm-adjusted p-values were used to account for multiple comparisons. Specifically, we examined semantic predictability by contrasting Congruent and Low Cloze, and Congruent and Incongruent endings within each of the four groups, and phonological facilitation by contrasting Pseudohomophone and Orthographic control endings within each group. Furthermore, to assess potential group differences in reading times, we conducted pairwise group comparisons within each Type of Ending using estimated marginal means, with p values adjusted for multiple comparisons (Holm). We also ran direct between-group comparisons (Holm corrected) of the three planned contrasts to assess the difference in magnitude of the effects.

Since we expected a significant phonological facilitation effect during sentence reading in hearing native speakers but not in the other groups, we complemented the a-priori contrasts for the phonological effect with Bayesian paired t-tests for the Deaf, Deaf CI, and HNN groups. We used the BayesFactor package in R (Morey et al., 2018) to run paired Bayesian t-tests over the log-transformed RTs (ttestBF, paired = TRUE) comparing Pseudohomophone vs. Orthographic control endings^4^, which allowed us to quantify the strength of evidence for or against a phonological effect within each group. We report Bayes factors in favour of the null over the alternative (BF_01_), where values > 1 indicate evidence for the null. For instance, for a given effect BF_01_= 4.2, the null hypothesis would be 4.2 times more likely than the alternative with the given set of data. Evidence categories (anecdotal, moderate, strong, decisive) followed the Jeffreys/Lee–Wagenmakers interpretive scale (Ly et al., 2016) where values between 1 and 3 are considered anecdotal, 3 to 10 moderate, 10 to 30 strong, and above 30 decisive.

### 3.1 Semantic effect.

There were significant differences between the Congruent and Incongruent conditions for each of the groups separately (all *p*s < .04; see table 2 for details and supplementary materials for the same results with the larger groups) revealing that reading times were faster for Congruent than Incongruent words for all groups. There were significant differences between the High Cloze (Congruent) and Low Cloze conditions for Deaf, HNN, and HN readers (all *p*s < .01; see Table 2 for details and supplementary materials for the same results with the larger groups), revealing that reading times were faster for High Cloze (Congruent) than Low Cloze words. The difference was not significant for Deaf-CI readers (*p* = .185). For the Deaf-CI group, we ran an exploratory analysis excluding participants that were not current CI users and those implanted at or after age 9 yrs. (n = 13). Holm-adjusted planned contrasts showed that Congruent endings were read significantly faster than Incongruent endings, *b* = −50.70 ms, *SE* = 16.80, *z* = −3.02, *p* = .008. The difference between congruent High Cloze and Low Cloze endings was not significant, *b* = −38.80 ms, *SE* = 19.80, *z* = −1.96, *p* = .101 in this exploratory analysis either.

Pairwise comparisons revealed no significant differences in reading times between participant groups for any Type of Ending after Holm correction for multiple comparisons (all *p*s > .8). Furthermore, direct between-group comparisons of both planned contrasts showed that the magnitude of the semantic congruency effect (Congruent vs. Incongruent) was larger for the HN than for the Deaf-CI group, *b* = 56.192, SE = 19.9, z = 2.83, *p* = .028. No other between-group comparisons reached significance after correction (all *p*s ≥ .05). There were no significant differences between the groups in the magnitude of the semantic prediction effect (Congruent vs. Low Cloze) (all ps = 1.000).

### 3.2 Phonological facilitation effect.

Only the HN group read the Pseudohomophones faster than the Orthographic control targets (*p* <.001; see Table 2 for details and supplementary materials for the same analyses and results with the larger groups). The paired Bayesian t-tests comparing Pseudohomophone vs. Orthographic control endings revealed that Deaf readers (BF_01_ = 4.20, median δ = 0.083, 95% HDI [−0.291, 0.458]), Deaf CI users (BF_01_ = 4.65, median δ = −0.006, 95% HDI [−0.391, 0.365]), and HNN (BF_01_ = 3.89, median δ = 0.113, 95% HDI [−0.277, 0.482]) showed moderate evidence for the null.

There were no significant differences in reading times between participant groups for any Type of Ending after Holm correction for multiple comparisons (all *p*s = 1). The magnitude of the phonological effect approached significance for the contrasts between HN and Deaf, *b* = 65.46 ms, SE = 24.7, z = 2.65, *p* = .054, Deaf-CI, *b* = 62.33 ms, SE = 24.8, z = 2.51, *p* = .060, and HNN readers, *b* = 58.95 ms, SE = 24.8 z = 2.37, *p* = .069^5^. For the Deaf-CI group, the exploratory analysis excluding participants that were not current CI users and those implanted at or after age 9 yrs. showed no significant differences between pseudohomophones and orthographic-control pseudowords, *b* = −13.70 ms, *SE* = 17.40, *z* = −0.79, *p* = .433.

### 3.3 Correlations with language skills.

We did not find any evidence of a correlation between the semantic or phonological effects (operationalised as congruent – Incongruent, High Cloze Congruent – Low Cloze, and Pseudohomophone – Orthographic Control RTs) and the linguistic skills measures, including reading ability, for any of the groups (all r < .266, all *p*s >.133). Table 3 displays the r and p values for each group.

## 4 Discussion

We examined semantic and phonological facilitation effects, using a self-paced sentence reading task, in four groups of readers (deaf readers without cochlear implants, deaf readers with cochlear implants [Deaf-CI], hearing native readers of English [HN], and hearing non-native [HNN] readers of English) matched on reading ability and English vocabulary. The results revealed three main findings. First, all groups showed robust semantic congruency effects, with slower reading times for incongruent than for congruent endings. Secondly, semantic predictability effects were observed in all groups except the Deaf-CI readers, whose pattern suggested strong integration but weaker prediction. Finally, the phonological effect was found only in the HN group. We now discuss the implications of this pattern for prediction accounts and for understanding sentence reading in deaf adults.

### 4.1 Semantic prediction

All four groups showed clear semantic congruency effects, with significantly longer reading times for incongruent than congruent sentence endings. In addition, Deaf, HN and HNN readers showed robust semantic prediction effects, with significantly faster reading times for the congruent endings in high- than low-predictability sentences. In contrast, the Deaf-CI group did not show this advantage in the main analysis, suggesting weaker semantic prediction that was not accounted for by age of implantation or CI use.

The findings for the Deaf, HN and HNN groups align with previous research showing that semantic prediction is a core component of sentence comprehension in both L1 and L2 readers (e.g., Payne & Federmeier, 2017; Roland et al., 2012). Together with the absence of group differences in condition-level reading times, this result extends recent evidence that skilled deaf adult readers can extract semantic information from written words on a time course similar to hearing readers, despite differences in language experience (Rowley et al., 2025). Notably, deaf participants showed this reading profile across a broad range of reading abilities, rather than only among highly skilled readers.

The findings of strong semantic congruency but weaker semantic prediction in the Deaf-CI readers were unexpected considering previous research. Auditory comprehension studies have found prediction of upcoming sentence completions in children with CIs at levels similar to hearing peers (Holt et al., 2021), and strong semantic mismatch responses in adults with CIs, interpreted as reflecting increased top-down semantic prediction to compensate for degraded bottom-up input (Kallioinen et al., 2023; Moberly & Reed, 2019). However, the present results are somewhat consistent with evidence of delayed reduction in processing effort for predictable sentences in adults with CIs (Winn, 2016). We extend these findings from the auditory domain to reading. One possibility is that reduced exposure to auditory input limits implicit learning of co-occurrence statistics, thereby constraining efficient use of sentence context (Pisoni et al., 2016). Importantly, however, the main analyses show that comparable reading skill, sentence comprehension, and vocabulary can be achieved through different predictive strategies.

Contrary to some previous reports of stronger predictability effects in skilled readers (Ng et al., 2017; Steen-Baker et al., 2017) or in slow readers (Ashby et al., 2005; Slattery & Yates, 2018), we did not observe a gradient in semantic effects as a function of individual differences in any of the language measures (i.e., the effect was significant after controlling for item- and participant-level covariates and no correlation was found between the semantic effects and the language skills). This may reflect the relatively simple sentence materials used in the current study. Future work should test whether differences in semantic prediction emerge with more demanding sentence contexts.

### 4.2 Phonological facilitation

First, the phonological effect was observed only in the HN group. Hearing native readers showed faster reading times for pseudohomophones than orthographic controls, consistent with prior evidence for preactivation of word form under highly predictive conditions (DeLong et al., 2005; Ito et al., 2016; Rayner, Pollatsek, & Binder, 1998). As in Rayner et al. (1998), it is likely that HN readers relaxed orthographic verification, allowing spelling discrepancies to initially go undetected when the pseudohomophone’s sound satisfied strong top-down expectations. Importantly, this phonological effect remained significant after controlling for reading ability, vocabulary, and item-level variables such as base-word frequency, suggesting that strong contextual constraint can facilitate the processing of visual input that matches the expected word phonologically, even when it mismatches orthographically.

Second, and crucially, no phonological facilitation effect was found in the deaf groups or in HNN readers despite matched comprehension and reading ability, suggesting that phonological facilitation is not a universal feature of sentence comprehension and depends on language experience and access to well-specified phonological representations. While we acknowledge the relatively small sample size (n = 24), Bayesian analyses provide evidence against the presence of a robust phonological facilitation effect emerging in a larger sample. These findings are consistent with Daneman and Reingold’s (2000) proposal that the dominant route from print to meaning can proceed directly from orthographic representations to semantics, with phonological activation being weaker and/or delayed. On this view, a printed form may eventually activate a phonological code, but this activation may not be sufficiently rapid or strong to influence reading times when phonological representations are less specified or less automatically engaged. Thus, for deaf and HNN readers, pseudohomophones may not provide the same processing advantage as in HN readers because the match between the target and the expected phonological form may not be available early or strongly enough to affect reading times. Instead, these readers may rely more strongly on orthographic-semantic mappings, detecting the mismatch between the printed input and the predicted word form rather than being facilitated by its sound. This interpretation is also consistent with evidence that phonological processing is not reliably associated with reading skill in deaf adults (Emmorey, 2020; Gutierrez-Sigut et al., 2017, 2018). More broadly, our findings extend recent work showing that deaf readers can achieve skilled comprehension and skip predictable words during reading while relying on strategies guided by visual properties of words, such as word length (Cooley et al., 2025). Thus, deaf readers can access and integrate word meaning on a time course comparable to hearing readers, achieving similar comprehension without showing a phonological facilitation in the present task. This interpretation is consistent with Thierfelder et al. (2020), who found that Hong Kong deaf readers of Chinese relied primarily on orthographic information during sentence reading. In their eye-tracking study, deaf readers showed robust orthographic facilitation in first fixation and gaze duration, whereas homophonic facilitation was not found in early measures. Their findings, like the present results, suggest that even if deaf readers activate phonological information in some circumstances, such activation is weaker, more delayed, and more dependent on context than in hearing readers. However, reduced spoken-phonological facilitation does not necessarily imply a total absence of form-based activation. Written words can also activate sign-language representations. Saunders et al. (2025) found early facilitation from phonologically related ASL translations during English sentence reading, particularly among deaf less-skilled readers. Likewise, Lan et al. (2026) found that spoken-phonological and sign-language facilitation varied with oral- and sign-language experience, respectively, and were further moderated by reading fluency. ERP evidence similarly indicates that spoken-, sign- and orthographic-form information can be activated at different processing stages in deaf readers (Thierfelder, 2024). Together, these findings suggest that the form-based representations recruited during reading depend on language experience, reading skill, script and processing stage, rather than reflecting a simple presence or absence of phonological activation.

Third, the absence of a phonological effect in Deaf-CI readers is particularly informative. Although CI use increases access to the auditory component of speech, the Deaf-CI readers did not score significantly higher than deaf non-CI readers in phonological decoding skill, nor did they show evidence of a phonological effect during sentence reading. This indicates that increased access to auditory information does not necessarily translate into automatic phonological processing. However, their comprehension was comparable to that of hearing readers, consistent with previous reports that CI users can achieve functional literacy (Mayer & Trezek, 2018, 2024), indicating that good comprehension can be achieved without phonological facilitation in this task. The null phonological effect did not appear to be related to age of implantation, which ranged from 1 to 31 years; the results remained unchanged when age of implantation was included in the model, and age of implantation did not correlate with the size of the phonological effect. One possibility is that participants used older implant technology, as most had their implants fitted in the late 1990s and early 2000s, which might have been less effective. Alternatively, CI individuals may have developed a route to reading comprehension that does not depend strongly on phonological processing. Further work is needed to characterise the precise mechanisms they use for reading comprehension.

Similarly, the lack of a phonological effect in HNN readers aligns with evidence that L2 readers often do not engage in form prediction (Ito, 2024; Ito et al., 2017b; Ito & Pickering, 2021; Ito et al., 2018). This finding is particularly notable given that HNN readers showed phonological decoding skills comparable to those of HN readers, suggesting that explicit phonological skill alone may not guarantee rapid phonological engagement during sentence reading. Instead, L2 readers may rely more on semantic and orthographic information. L2 readers may also face cross-language interference from co-activated L1 words (Chambers & Cooke, 2009; Shook & Marian, 2013) or rely more heavily on lexical-semantic cues than on detailed syntactic representations (e.g., Clahsen & Felser, 2006). Such processing demands may make sentence interpretation more effortful, leaving fewer resources available for preactivating phonological form.

Importantly, because pseudohomophones were included only in high-cloze sentences, the present design cannot fully dissociate between two possible sources of facilitation. One possibility is that semantic expectations preactivate compatible phonological representations before target onset. Another is that facilitation emerges during the recognition and integration of the pseudohomophone itself because the pseudohomophone looks like a real word. However, a purely postlexical word-likeness account seems insufficient. HN readers showed clear evidence of semantic prediction during reading, as high-cloze congruent endings were read faster than low-cloze congruent endings, and the orthographic controls were also pronounceable and word-like, with a similar Levenshtein distance to a real word as the pseudohomophones. The selective advantage for pseudohomophones over orthographic controls in HN readers is therefore more consistent with context-driven activation of phonological representations, such that bottom-up input matching the predicted phonology is processed more efficiently even when the spelling mismatches the expected word. Importantly, regardless of whether the HN effect arises before target onset or during early recognition and integration, the group contrast remains clear: phonological overlap with the predicted word did not facilitate processing in the deaf or HNN groups. This provides evidence against robust phonological facilitation in these readers and supports the view that phonological facilitation during sentence reading depends on native-like, automatically engaged phonological representations, rather than being a universal consequence of contextual constraint.

Deaf readers, particularly those without cochlear implants, showed lower explicit phonological skill and relatively stronger orthographic skill, yet achieved comparable comprehension. Moreover, the absence of a phonological effect in HNN readers suggests that explicit phonological knowledge alone is not sufficient to produce phonological facilitation during sentence reading. What appears to matter is not simply whether readers can access phonology, but whether phonological representations are native-like, automatic, and rapidly available during reading. The present findings therefore indicate that in deaf adults neither good sentence comprehension nor semantic facilitation during reading depends on strong phonological representations or on the online use of phonological information. Despite lower performance on explicit speech-phonology manipulation tasks and the absence of a phonological effect, deaf readers without CIs showed robust semantic prediction and comprehension accuracy comparable to hearing native readers. Rather than constituting a bottleneck, reduced phonological specificity may therefore be compensated for by robust orthographic processing (Emmorey & Lee, 2021; Gutierrez-Sigut et al., 2019). Consistent with this view, both deaf groups outperformed the HN group in orthographic decoding, suggesting that orthographic processing may support comprehension in deaf readers. However, this account does not explain the weaker semantic prediction observed in the Deaf-CI group, and orthographic decoding skill did not modulate the semantic effect in any group. The role of orthographic processing in sentence comprehension therefore requires further investigation, ideally using orthographic tasks that are more challenging for deaf adults and allow greater variability in performance.

Notably, although both orthographic and phonological decoding tasks correlated with overall reading ability across groups, neither correlated with the size of the semantic or phonological effects, even in the larger samples reported in the Supplementary Materials. This raises the possibility that these explicit tasks do not capture the automatic processes involved in self-paced reading comprehension (for discussion, see Rowley et al., 2025). Future research should therefore clarify how metalinguistic skills contribute to real-time reading processes and reading ability more generally, potentially by incorporating richer measures of implicit processing, such as online EEG recordings.

Taken together, the present findings indicate that similar comprehension outcomes can be achieved through different processing routes, depending on the reader’s linguistic profile.

### 4.3 Implications for prediction theories

Overall, our findings support models of prediction as a flexible, hierarchical mechanism that draws on multiple sources of information beginning with semantic and subsequently cascading to phonological activation if the right conditions are met (Burnsky, 2022; Federmeier, 2022; Huettig, 2015; Pickering & Gambi, 2018). Semantic prediction was robust across Deaf, HN, and HNN groups and approached significance in the whole-sample Deaf-CI group. Hence, most readers anticipate upcoming semantic content either through a general, low-resource, automatic spreading activation between related concepts in memory (Federmeier, 2022; Pickering & Gambi, 2018), or because the high predictability (low surprisal) of the target word reduces the cognitive effort required for word access and semantic integration in the sentence context (see, e.g.,, Wilcox et al., 2023).

In contrast, the phonological effect appears to be an optional mechanism that is engaged when readers possess sufficient cognitive and linguistic resources and when processing time is adequate (Pickering & Gambi, 2018; for review see Wong et al., 2025). Note that, because participant-controlled word exposure in the self-paced reading task, the absence of phonological facilitation in deaf and HNN readers is unlikely to reflect insufficient exposure time. Regarding differences in linguistic resources, because the groups were matched on reading skill and vocabulary, it is unlikely that the observed group differences can be attributed to these variables. HN readers demonstrated higher phonological skill than deaf readers but did not differ significantly from HNN readers. However, despite comparable phonological skills to the hearing HN readers, HNN readers did not exhibit phonological facilitation effects. This indicates that lower phonological skill alone in deaf readers does not fully account for the lack of phonological facilitation.

### 4.4 Limitations

While the sample sizes for the matched groups were not very large, Bayesian analyses helped mitigate concerns about statistical power by quantifying evidence for the absence of effects. Nonetheless, future studies with larger, more diverse samples are needed to further investigate individual differences in semantic prediction and phonological facilitation. We acknowledge the substantial variability within the groups studied, such as differences in age of implantation among Deaf-CI readers and diversity of first-language backgrounds among HNN readers. This variability in language and audiological background reflects the natural heterogeneity of these populations. Importantly, the reported effects remain after controlling for a range of relevant participant- and item-level variables and, for the Deaf-CI group, restricting the analysis to current users who were implanted by age 9yrs. At the same time, this heterogeneity limits the extent to which strong general conclusions about processing mechanisms can be drawn. Larger-scale studies that include additional factors not examined here, such as detailed histories of hearing-aid use, would be better suited to addressing such broader generalisations. For example, future studies are needed to disentangle implantation age from implant benefit, duration of use, or participants’ speech-language and educational histories.

In this study we operationalised semantic prediction as faster reading of expected high-cloze words than less predictable or incongruent words and phonological prediction as faster reading times of pseudowords with phonological overlap with the highly predictable word than to unrelated (orthographic control) pseudowords. These measures are compatible with prediction but do not easily distinguish pre-target preactivation from easier lexical access due to context, reduced surprisal or facilitated post-lexical integration. Future studies should examine whether targets semantically related but non-identical to the highly predicted ending are facilitated relative to an unrelated target or should use a measure sensitive to anticipatory processing before target onset, such as eye tracking or EEG measures.

It is possible that sentence-level processes may have contributed to the observed effects. Sentence wrap-up effects, typically reflected in longer reading times at the end of a sentence (Meister et al., 2022), have been shown to be reduced in lower-skill readers (Ng et al., 2020) and to increase when readers integrate complex or ambiguous information (Payne & Stine-Morrow, 2014; Rayner et al., 2000; Warren et al., 2009). Given the high comprehension accuracy and the similarity in reading times across conditions and groups, there is little evidence that deaf and HNN readers engaged differently in sentence wrap-up processes compared with HN readers. Nonetheless, we acknowledge that the present design may not have captured subtle wrap-up differences, as the sentences were neither complex nor ambiguous. Indeed, sentences in the congruent and low-cloze conditions were highly predictive and/or simple, with a Flesch–Kincaid score of 4.1, corresponding to an elementary-school reading level (ages 9–10). In the remaining conditions, reading times were compared across identical sentence contexts followed by either an incongruent word or one of two types of pseudowords (pseudohomophone or orthographic control). These endings should be equally difficult to integrate for all groups, unless the phonology of the predicted word is processed online, as observed in the pseudoword condition for HN readers.

Another possibility is that phonological effects in deaf and HNN readers were too weak to emerge at the target word and instead would have appeared in spillover regions (Reichle et al., 2003; Reilly & Radach, 2006). However, spillover effects are typically associated with later stages of sentence comprehension, such as integration and validation (Rayner & Duffy, 1986), whereas the present study focused on early facilitation effects, which are most likely to be observed at the target word in highly predictable contexts. Although phonological facilitation in deaf and HNN readers could potentially emerge with longer sentence continuations, our data show that such effects were consistently weaker than both the semantic effects within these groups and the phonological effect observed in HN readers.

Future research should explore whether phonological facilitation effects might emerge under different task conditions (e.g., slower presentation rates, more complex sentences, or extended sentence regions), or when using more sensitive techniques such as event-related potentials.

## 5 Conclusions

The present study shows that the effect of semantic contextual prediction during reading is robust across most reader groups and does not depend on access to speech phonology. In contrast, phonological facilitation was observed only in hearing native readers, suggesting it is not a universal feature of comprehension. These findings support the view that skilled reading can be achieved through different routes, depending on language experience, with phonological prediction emerging only as a result of specific language experience.

## Supplementary Material

Supplementary Material

## Figures and Tables

**Table 1:** Average reading times (in ms) and SD for each group and condition.

	Deaf		Deaf CI		HN		HNN	
**Condition**	**Average**	**SD**	**Average**	**SD**	**Average**	**SD**	**Average**	**SD**
Congruent	581.1	270	544.7	267	533.1	269	596.0	299
Incongruent	630.0	322	570.8	285	579.5	317	643.3	344
Low Cloze	677.6	378	591.5	355	646.2	407	656.3	376
Orthographic Control	653.9	375	589.8	346	652.9	400	653.6	371
Pseudohomophone	653.3	381	579.1	334	584.7	331	641.4	354

**Table 2:** Detailed statistics, Holm-adjusted *p* values for the contrasts of interest in each group (significant contrasts in bold).

Contrast	Group	Estimate	SE	Confidence Interval 95% Lower	Confidence Interval 95% Upper	z	p
**Congruent vs. Incongruent**							
	**Deaf**	**−84.17**	**14.7**	**−119.30**	**−49.03**	**−5.735**	**< .0001**
	**Deaf CI**	**−35.09**	**13.8**	**−68.10**	**−2.12**	**−2.548**	**.0325**
	**HNN**	**−49.45**	**14.1**	**−83.30**	**−15.62**	**−3.500**	**.0014**
	**HN**	**−91.28**	**14.2**	**−125.30**	**−57.23**	**−6.418**	**< .0001**
**High Cloze (Congruent) vs. Low Cloze**	**Deaf**	**−42.38**	**14.6**	**−77.30**	**−7.43**	**−2.903**	**.0074**
	Deaf CI	−24.77	14.8	−60.20	10.67	−1.673	.1885
	**HNN**	**−42.73**	**15.1**	**−78.90**	**−6.53**	**−2.826**	**.0094**
	**HN**	**−41.86**	**13.7**	**−74.60**	**−9.16**	**−3.065**	**.0022**

**Phonological**	Deaf	−3.30	17.6	−45.50	38.92	−0.187	.8517
	Deaf CI	− −6.07	17.5	−47.90	35.81	−0.347	.7288
	HNN	−9.44	17.6	−51.50	32.59	−0.538	.5907
	**HN**	**−68.39**	**17.6**	**−110.60**	**−26.22**	**−3.882**	**.0002**

**Table 3:** Correlations, all non-significant, between the semantic and phonological effects and the reading-related skills

		Deaf		Deaf CI		HNN	HN		
		r	p	r	p	r	p	r	p
High Cloze Congruent vs. Incongruent	**VW**								
		-0.041	0.849	-0.001	0.995	0.117	0.587	0.127	0.555
	**LEXTALE**	-0.162	0.46	0.017	0.937	0.034	0.876	0.22	0.302
	**Phonological coding**	-0.232	0.276	0.067	0.755	-0.173	0.419	0.32	0.147
	**Orthographic coding**	-0.198	0.355	-0.316	0.133	0.098	0.649	0.335	0.127
		0.196	0.359	-0.008	0.971	0.266	0.209	-0.038	0.86
High Cloze Congruent vs. Low Cloze	**VW**	0.048	0.829	-0.061	0.776	0.139	0.517	-0.068	0.754
	**LEXTALE**	0.204	0.339	-0.038	0.858	-0.117	0.585	0.102	0.651
	**Phonological coding**	0.224	0.293	-0.252	0.236	0.236	0.267	0.088	0.696
	**Orthographic coding**	-0.058	0.787	0.101	0.639	-0.09	0.675	0.282	0.182
		0.068	0.757	-0.074	0.731	0.048	0.824	0.213	0.317
		-0.053	0.804	-0.006	0.976	0.028	0.897	0.086	0.703
Phonological effect	**VW**								
		-0.085	0.695	0.079	0.713	0.03	0.89	0.209	0.35
	**LEXTALE**	-0.041	0.849	-0.001	0.995	0.117	0.587	0.127	0.555
	**Phonological coding**	-0.162	0.46	0.017	0.937	0.034	0.876	0.22	0.302
	**Orthographic coding**	-0.232	0.276	0.067	0.755	-0.173	0.419	0.32	0.147

## Data Availability

All data, materials, and analysis code supporting the findings of this study are openly available on the Open Science Framework (OSF): https://osf.io/8kfz6/.

## Highlights

Semantic prediction was robust across all reader groups

Only hearing native users of English showed phonological facilitation

Deaf readers engaged semantic but not phonological prediction

Cochlear implants did not boost phonological facilitation

Reading skill was not linked to prediction strength
