# Supplementary material for: Predictive Processing in Sentence Reading: Semantic prediction but not phonological facilitation in deaf readers
Source: Cognition. Author manuscript; Available in PMC 2026 Nov 16. (PMC7619737; doi:10.1016/j.cognition.2026.106703)
Supplement: Supplementary file 1 [file EMS218932-supplement-Supplementary_Material.docx]

**Predictive Processing in Sentence Reading: Semantic prediction**

**but not phonological facilitation in deaf readers**

Gutierrez-Sigut E, Perea, M., Vergara-Martinez, M., MacSweeney, M., Rowley, K.,

Saenz, I.

**Supplementary materials**

**Appendix A. Whole sample analyses.**

Data are presented below for the entire sample of participants, not only those matched on the key variables reported in the main study. In summary, the patterns are the same for the larger unbalanced and unmatched samples as described in the matched groups.

**Participants**

*Deaf*. The deaf group comprised the same 24 participants reported in the main text.

Deaf CI. Fifty-one deaf participants (23 female, 27 male, and 1 non-binary) with a Cochlear implant (CI) also participated in this study. Their average age was 25.4 (SD = 4.9; range 20 to 42). Participants reported being severely or profoundly deaf before age 3, except for one who reported having been diagnosed between ages 4 and 6, and having the CI fitted at age 5. Their age of implantation varied from 0 to 31 years of age, with 32 participants reporting having had an implant before age 9 yrs, five participants reported having their implant fitted after 12 yrs, and three participants did not answer this question. One participant did not know British Sign Language (BSL), 25 were native signers, 19 were early signers (BSL AoA between 4 and 8 years old), 3 were late signers (BSL AoA 9 years old and older), and 3 did not report when they started to sign. Participants were recruited through invitation due to having participated in previous research and via researchers’ personal contacts.

*Hearing Native* (HN). One hundred and eleven (72 female, 37 male, 2 prefer not to say) hearing participants who were early readers of English also participated in this study. Their average age was 33.1 (SD = 10.4; range 19 to 55). Participants were recruited through prolific and via the participant’s database at the University of Essex.

*Hearing Non-native* (HNN). Eighty-three hearing participants (58 female, 25 male) who were non-native speakers of English and therefore read in their second language were also recruited for this experiment. All non-native participants were proficient in English and had passed the required English exams to be admitted to the university. Their average age was 35.5 (SD = 14.4; range 19 to 63). Twenty-four participants reported Mandarin Chinese as their first language, and fifteen reported Cantonese. The remaining participants reported languages such as Albanian, French, German, Guangdong, Hindi, Italian, Konkani, Lithuanian, Malayalam, Memoni, Norwegian, Polish, Portuguese, Romanian, Spanish, Swahili, and Urdu as their first language. Seven learned English between 0 and 2 yrs, forty between 3 and 5 years, eighteen between 6 and 8 yrs, eight between 9 and 11 years, 5 between 12 and 15 years, and finally 5 were over 15 yrs when they learned English. All reported to have learnt English as an L2 in restricted environments/hours of the day while immersed in their L1 environment. All Participants were recruited through Prolific and via the participant’s database at the University of Essex.

ANOVAs for reading skill, vocabulary-based English proficiency, and phonological and orthographic skills [Anova [*skill of interest*] ~ Group, pstHocCorrectionHolm = TRUE, postHocTerms = ~ Group] showed that the HN scored higher in reading ability than the three other groups (all *p*s < .024), the Deaf, Deaf CI, and HNN groups were not significantly different (all *p*s > .1)

The HNN group had a significantly lower score in the LEXTALE score than the HN group (t = -3.05, *p* = .015), but there were no other significant differences between the groups.

|  | Deaf | | Deaf_CI | | HNN | | HN | |
| --- | --- | --- | --- | --- | --- | --- | --- | --- |
|  | Average | SD | Average | SD | Average | SD | Average | SD |
| VW | 21.5 | 8.8 | 21.2 | 7.4 | 19.8 | 6.7 | 26.5 | 7.9 |
| LEXTALE | 78.6 | 18.9 | 74 | 17.5 | 68.9 | 17.9 | 78.8 | 23.8 |
| Phonological skill | 60.1 | 21.5 | 63.9 | 13.2 | 85 | 18.1 | 94.8 | 7.1 |
| Orthographic skill | 94.7 | 9.6 | 95.7 | 4.7 | 55.5 | 23.2 | 79.5 | 20.7 |

Regarding phonological decoding, the Deaf and Deaf CI group did not have a significant difference (t = -1.098, *p* = 1.000) but both had lower scores than the HNN and the HN (all *p*s < .001). The HNN also had significantly lower scores than the HN group (t = -4.418, *p* < .001).

Finally, regarding orthographic skill, the Deaf and Deaf CI group did not have a significant difference (t = -0.235, *p* = 1.000), but both groups of deaf participants had higher scores than the HNN and the HN (all *p*s < .002). The HN group had a higher score than the HNN group (t = -8.021, *p* < .001).

Reading ability was correlated with vocabulary based English proficiency and with phonological skills in all groups (all *p*s < .028), and with orthographic skill in the Deaf, Deaf CI and HN groups (all *p*s < .033), but not in the HNN group (*p* = .086) (See Table S1)

Table S1.

|  | Deaf | | Deaf CI | | HNN | | HN | |
| --- | --- | --- | --- | --- | --- | --- | --- | --- |
|  | r | p | r | p | r | p | r | p |
| LEXTALE | 0.679 | < .001 | 0.274 | 0.028 | 0.325 | 0.005 | 0.36 | < .001 |
| Phonological  decoding | 0.669 | < .001 | 0.571 | < .001 | 0.313 | 0.005 | 0.378 | < .001 |
| Orthographic  decoding | 0.382 | 0.033 | 0.356 | 0.006 | 0.176 | 0.086 | 0.461 | < .001 |

**Results**

RTs below 250 ms and above 2500 ms (3.6%) were excluded from the analyses. Since the incongruent, pseudohomophone, and orthographic control conditions were designed to be not fully comprehensible, no RTs were excluded due to errors in the comprehension questions (see Keating & Jegerski, 2015). All participants engaged with the task and answered the comprehension question accurately, the average accuracy was 93.2% (SD 5.8) correct (Deaf = 92.9%, Deaf CI = 93.8%, HN = 93.4%, and HNN = 90.7%). Table S2 details the average reading times for each condition and group (see also Figure S1).

Table S2. Average reading times for each group and condition.

|  | Deaf | | Deaf CI | | HN | | HNN | |
| --- | --- | --- | --- | --- | --- | --- | --- | --- |
| Condition | Average | SD | Average | SD | Average | SD | Average | SD |
| Congruent | 581.1 | 270 | 537.6 | 269 | 508.7 | 254 | 600.3 | 301 |
| Incongruent | 630 | 322 | 558.7 | 279 | 541.3 | 278 | 645 | 329 |
| Low Cloze | 677.6 | 378 | 587.7 | 358 | 607.6 | 389 | 695.5 | 415 |
| Orthographic Control | 653.9 | 375 | 582.0 | 343 | 603.2 | 378 | 689 | 392 |
| Pseudohomophone | 653.3 | 381 | 578.8 | 339 | 559.0 | 331 | 687.6 | 381 |

To analyse the reading times to the targets, we fitted a generalized linear mixed‑effects model (GLMM), in R (R Core Team, 2021) (version 4.4.1) using the glmmTMB package (Brooks et al., 2017). The model used a Gamma distribution with a log link to account for the positive skew of RT distributions (Lo & Andrews, 2015). We used the most complex model random-effects structure that converged (RT ~ Group * Type of ending + Age + LEXTALE + VW + Length + ON density + ON frequency + (1 + Type of ending || ID) + (1 | itemID)) following recommendations for maximal random-effects structures (Barr et al., 2013). The random-effects structure included random intercepts and slopes for participants. Age, as well as LEXTALE and Vernon-Warden scores, were added as participant-level covariates to account for potential differences in age distribution, vocabulary, and reading skill between the groups. Length, orthographic neighbourhood density, and orthographic neighbourhood frequency were added to model systematic variability, while random intercepts for items captured residual item‑to‑item variability. To test our hypothesis, we specified planned contrasts using the emmeans package (Lenth, 2023), estimated marginal means were computed on the response (reading‑time) scale, Holm-adjusted p-values were used to account for multiple comparisons. Specifically, we examined semantic preactivation by contrasting Congruent and Low Cloze, and Congruent and Incongruent endings within each of the four groups, and phonological preactivation by contrasting Pseudohomophone and Orthographic control endings within each group. To assess potential group differences in reading times, we conducted pairwise group comparisons within each Type of Ending using estimated marginal means, with p values adjusted for multiple comparisons (Holm). We also run direct between‑group comparisons (Holm-corrected) of the three planned contrasts to assess the differences in effect magnitude.

Since we expected a significant effect of phonological preactivation during sentence reading in hearing native speakers but not in the other groups, we complemented the a-priori contrasts with Bayesian paired t‑tests for the Deaf, Deaf CI, and HNN groups. We used the BayesFactor package in R (Morey et al., 2018) to run paired Bayesian t-tests over the log-transformed RTs (ttestBF, paired = TRUE) comparing Pseudohomophone vs. Orthographic control endings^[1]^, which allowed us to quantify the strength of evidence for or against a phonological effect within each group. We report Bayes factors in favour of the null over the alternative (BF₀₁), where values > 1 indicate evidence for the null. For instance, for a given effect BF₀₁= 4.2, the null hypothesis would be 4.2 times more likely than the alternative with the given set of data. Evidence categories (anecdotal, moderate, strong, decisive) followed the Jeffreys/Lee–Wagenmakers interpretive scale (Ly et al., 2016) where values between 1 and 3 are considered anecdotal, 3 to 10 moderate, 10 to 30 strong, and above 30 decisive.

**Semantic effect**. Reading times were faster for Congruent than Incongruent words for all groups (all *p*s < .001; see Table S3 for details). There were significant differences between the Congruent and Low Cloze conditions for the Deaf, HNN, and HN groups (all *p*s < .01), and the difference approached significance for the Deaf-CI readers (*p* = .051), revealing that reading times were faster for Congruent than Low Cloze words. For the Deaf-CI group, we ran an exploratory analysis excluding participants that were not current CI users and those implanted at or after age 9 (n = 24). Holm-adjusted planned contrasts showed that Congruent endings were read significantly faster than Incongruent endings, *b* = −45.47 ms, *SE* = 12.05, *z* = −3.77, *p* < .001. The difference between congruent High Cloze and Low Cloze endings was not significant, *b*= −23.03 ms, *SE* = 12.70, *z* = −1.81, *p* = .140.

HN participants were faster than Deaf‑CI participants in the Congruent (*p* = .017), and Low‑cloze (*p* = .041), conditions, and faster than HNN participants in the Congruent (*p* = .0003), Incongruent (*p* = .002), and Low‑cloze (*p* = .0002) conditions. All other between‑group comparisons were nonsignificant (all *ps* ≥ .35). In addition, Deaf‑CI participants showed a smaller Congruent–Incongruent semantic effect than both HN (*p* = .006) and HNN participants (*p* = .021); no other between‑group differences in effect size were observed (all *ps* ≥ .054).

**Phonological effect.** Only the HN group read the Pseudohomophones faster than the Orthographic control targets (p <.001; see Table S3 for details). For the Deaf-CI group, the exploratory analysis excluding participants that were not current CI users and those implanted at or after age 9 showed no differences between pseudohomophones and orthographic-control pseudowords, *b* = 6.78 ms, *SE* = 13.53, *z* = 0.50, *p* = .616. The paired Bayesian t-tests comparing Pseudohomophone vs. Orthographic control endings revealed that Deaf readers (BF₀₁ = 4.20, median δ = 0.084, 95% HDI [−0.303, 0.456]), Deaf CI users (BF₀₁ = 6.54, median δ = −0.013, 95% HDI [−0.280, 0.244]), and Hearing non-native English speakers (BF₀₁ = 7.87, median δ = −0.032, 95% HDI [−0.240, 0.181]) showed respectively moderate, substantial, and strong evidence in favour of the null hypothesis.

HN participants were faster than Deaf‑CI participants in the Pseudohomophone (*p* = .017) condition, and faster than HNN participants in the Orthographic Control (*p* = .002) and Pseudohomophone (*p* < .0001) conditions. All other between‑group comparisons were nonsignificant (all *ps* ≥ .35). In addition, Deaf‑CI participants showed a smaller phonological effect than HN participants (*p* = .007); no other between‑group differences in effect size were observed (all *ps* ≥ .054)^[2]^.

Table S3. Detailed statistics, Bonferroni-adjusted *p* values for the contrasts of interest in each group separately.

| Contrast | **Group** | **Estimate** | **SE** | **Confidence Interval 95% Lower** | **Confidence Interval 95% Upper** | **z** | **p** |
| --- | --- | --- | --- | --- | --- | --- | --- |
| High Cloze (Congruent) vs. Incongruent | **Deaf** | **−79.77** | **14.40** | **−114.20** | **−45.35** | **−5.548** | **< .0001** |
|  | **Deaf CI** | **−37.94** | **9.62** | **−61.00** | **−14.92** | **−3.945** | **.0002** |
|  | **HNN** | **−77.17** | **9.60** | **−100.10** | **−54.20** | **−8.043** | **< .0001** |
|  | **HN** | **−76.35** | **6.44** | **−91.80** | **−60.93** | **−11.855** | **< .0001** |
| High Cloze (Congruent) vs. Low Cloze | **Deaf** | **−40.55** | **13.90** | **−73.80** | **−7.36** | **−2.924** | **.0069** |
|  | **Deaf CI** | −24.85 | 11.10 | −51.50 | 1.75 | −2.236 | .0507 |
|  | **HNN** | **−40.96** | **11.00** | **−67.30** | **−14.62** | **−3.723** | **.0004** |
|  | **HN** | **−30.56** | **7.93** | **−49.50** | **−11.59** | **−3.856** | **.0001** |
|  |  |  |  |  |  |  |  |
| Phonological | Deaf | −1.06 | 17.80 | −43.70 | 41.56 | −0.060 | .9524 |
|  | Deaf CI | 5.30 | 12.50 | −24.60 | 35.19 | 0.424 | .6715 |
|  | HNN | −4.81 | 12.40 | −34.40 | 24.76 | −0.390 | .6968 |
|  | **HN** | **−42.18** | **7.88** | **−61.00** | **−23.31** | **−5.353** | **< .0001** |

**Figure S1.** Average reading times in milliseconds for the four experimental conditions: congruent (light grey), Low Cloze (dark grey), Incongruent (black), Orthographic control (blue) and pseudohomophone (orange) for each group separately (Deaf, Deaf CI, HNN, and HN). Error bars show SE.

Correlations with language skills (see Table S4 and figure S2). In the HNN group, orthographic coding was positively correlated with the Congruent–Low Cloze reading-time effect, r = .29, p = .020, and this association remained significant after excluding influential cases identified by Cook’s distance, leverage, and standardised residuals, r = .30, p = .024. In the HN group, phonological coding was initially positively correlated with the Congruent–Incongruent reading-time effect, r = .23, p = .024, but this association was no longer significant after excluding influential cases, r = .08, p = .441. There were no other significant correlations (all *p*s >.052).

Table S4. Correlations, all non-significant, between the semantic and phonological effects and the reading-related skills.

|  |  | Deaf | | Deaf CI | | HNN | | HN | |
| --- | --- | --- | --- | --- | --- | --- | --- | --- | --- |
|  |  | r | p | r | p | r | p | r | p |
| High Cloze (Congruent) vs. Incongruent | VW | -0.041 | 0.849 | 0.043 | 0.767 | 0.03 | 0.808 | 0.154 | 0.112 |
|  | LEXTALE | -0.162 | 0.46 | 0.147 | 0.308 | -0.025 | 0.844 | -0.113 | 0.251 |
|  | Phonological coding | -0.232 | 0.276 | -0.015 | 0.92 | -0.147 | 0.232 | **0.23** | **0.024** |
|  | Orthographic coding | -0.198 | 0.355 | -0.254 | 0.076 | 0.194 | 0.127 | -0.094 | 0.365 |
|  |  |  |  |  |  |  |  |  |  |
| High Cloze (Congruent) vs. Low Cloze | VW | 0.196 | 0.359 | -0.001 | 0.992 | 0.113 | 0.363 | 0.038 | 0.695 |
|  | LEXTALE | 0.048 | 0.829 | 0.093 | 0.52 | 0.106 | 0.398 | -0.102 | 0.297 |
|  | Phonological coding | 0.204 | 0.339 | 0.000 | 0.999 | -0.218 | 0.074 | 0.176 | 0.085 |
|  | Orthographic coding | 0.224 | 0.293 | -0.146 | 0.311 | **0.293** | **0.02** | -0.112 | 0.281 |
|  |  |  |  |  |  |  |  |  |  |
|  |  |  |  |  |  |  |  |  |  |
| Phonological effect | VW | -0.058 | 0.787 | 0.046 | 0.752 | -0.053 | 0.67 | 0.045 | 0.644 |
|  | LEXTALE | 0.068 | 0.757 | 0.065 | 0.655 | 0.221 | 0.075 | -0.061 | 0.535 |
|  | Phonological coding | -0.053 | 0.804 | -0.115 | 0.431 | -0.081 | 0.514 | 0.135 | 0.188 |
|  | Orthographic coding | -0.085 | 0.695 | 0.051 | 0.723 | -0.137 | 0.284 | -0.177 | 0.087 |

**Figure S2.** Scatterplots showing the significant correlations between the semantic effect and reading‑related skills.

**Appendix B. Whole sample analyses per group.**

Since some participants differ in other dimensions besides age, Vernon Warden, LEXTALE, and orthographic and phonological decoding skills (all included in the models in Appendix A), we run two separate models including group-specific variables that are unique to each group.

For the Deaf-CI group the model additionally included current CI users and the age at which their implant was fitted ((RT ~ Group * Type of ending + Age + Orthographic decoding + Phonological decoding + CI_use + Age_CI + Length + ON density + ON frequency + (1 + Type of ending || ID) + (1 | itemID)). In the Deaf‑CI group, reading times were faster to congruent than incongruent endings (*b* = −44.8 ms, *SE* = 11.0, *z* = −4.08, *p* = .0001). The Congruent–Low‑cloze contrast only approached significance (*b* = −27.8 ms, *SE* = 12.7, *z* = −2.19, *p* = .058). No phonological effect was observed (*b* = 1.5 ms, *SE* = 13.5, *z* = 0.11, *p* = .912).

To thoroughly test whether increased access to speech via the CI impacts the use of phonology during sentence reading we also examined the correlation between age at which the implant was fitted and the size of the phonological effect (see Figure S3). The correlation was far from significant (*r* = -0.006, *p* = .976), and visual exam of the scatterplot further supports the absence of a relationship for this group.

**Figure S3.** Scatterplot illustrating the absence of a correlation between age of implantation and the phonological effect.

For the HNN group, the model additionally included First Language and English Age of Acquisition (RT ~ Group * Type of ending + Age + Orthographic decoding + Phonological decoding English_AoA + First_Language + Length + ON density + ON frequency + (1 + Type of ending || ID) + (1 | itemID)). In the HNN group, we observed faster responses for Congruent than Incongruent endings (*b* = −87.9 ms, *SE* = 10.9, *z* = −8.08, *p* < .0001) and for Congruent than Low‑cloze endings (*b* = −45.8 ms, *SE* = 13.5, *z* = −3.38, *p* = .0015). No phonological effect was observed, with comparable response times for pseudohomophone and orthographic control conditions (*b* = −6.3 ms, *SE* = 15.0, *z* = −0.42, *p* = .674).

In sum, after controlling for residual variability associated with group heterogeneity, we observed the same pattern of effects than in the matched groups and the whole-group analysis in Appendix A. That is, weak semantic prediction in the Deaf‑CI group, strong semantic prediction in the HNN group, and an absence of phonological preactivation in both groups.

1. The tests used the default JZS prior (Cauchy) on the standardized effect size δ with the “medium” scale (r = √2/2 ≈ 0.707; Rouder et al., 2009) To quantify effect magnitude and uncertainty, we drew 20,000 posterior samples for δ using BayesFactor::posterior and summarized the posterior by the median and the 95% highest‑density interval (HDI), computed with the HD Interval package (hdi; Meredith & Kruschke, 2016)

   Meredith, M., & Kruschke, J. (2016). HDInterval: highest (posterior) density intervals. *CRAN: Contributed Packages*. ). Rouder, J. N., Speckman, P. L., Sun, D., Morey, R. D., & Iverson, G. (2009). Bayesian t tests for accepting and rejecting the null hypothesis. *Psychonomic Bulletin & Review*, *16*(2), 225-237.
2. Note that a model restricted to the Pseudohomophone and Orthographic control Types of Ending, including as a covariate the Levenshtein distance between the base word and either pseudoword ending (RT ~ Group * Type of ending + Age + Orthographic decoding + Phonological decoding + Length + ON density + ON frequency + LD + (1 + Type of ending || ID) + (1 | itemID)) yielded the same results except that the magnitude of the effect ws significantly larger for the HN than each of the three other groups (all *p*s < .05).
